# Absence of association between *SERPINE2 *genetic polymorphisms and chronic obstructive pulmonary disease in Han Chinese: a case-control cohort study

**DOI:** 10.1186/1471-2350-10-66

**Published:** 2009-07-16

**Authors:** Li Zhong, Wei-Ping Fu, Chang Sun, Lu-Ming Dai, Ya-Ping Zhang

**Affiliations:** 1 Laboratory for Conservation and Utilization of Bio-resource, Yunnan University, Kunming 650091, PR China; 2 Department of Respiratory Critical Care Medicine, The First Affiliated Hospital of Kunming Medical College, Kunming 650032, PR China; 3 State Key Laboratory of Genetic Resources and Evolution, Kunming Institute of Zoology, Chinese Academy of Sciences, Kunming 650223, PR China

## Abstract

**Background:**

Recent studies have proposed that the serine protease inhibitor E2 (*SERPINE2*) was a novel susceptibility gene for chronic obstructive pulmonary disease (COPD) in Caucasians. However, this issue still remained controversial. Additional evidences from populations with different environments and/or genetic backgrounds, such as East Asian, would be helpful to elucidate the issue.

**Methods:**

In this study, five proposed causal SNPs in *SERPINE2 *were genotyped in 327 COPD patients and 349 controls, all of which belonged to the Han population sampled from Southwest China. The frequency of each SNP was compared both individually and in combination between patients and controls. The potential relationship between these SNPs and severity of COPD was also investigated.

**Results:**

Three SNPs (rs3795877, rs6747096, and rs3795879) showed complete linkage disequilibrium (r^2 ^= 1), and the minor allele frequencies were 13.0% and 12.9% in case and control cohorts, respectively, with no significant difference observed (*P *= 0.96). We also failed to observe any significant correlation between these SNPs and COPD severity (*P *= 0.67). The other two SNPs (rs7579646 and rs840088) also presented a similar pattern. Moreover, four major haplotypes were observed in our sample but none showed a significant difference between case and control groups (*P *> 0.1).

**Conclusion:**

Our results failed to obtain the evidence that these SNPs in *SERPINE2 *contributed to the COPD susceptibility in the Han Chinese population.

## Background

Chronic obstructive pulmonary disease (COPD, MIM# 606963), one of the major sources of morbidity all over the world [1], is characterized by airflow limitation that is not fully reversible and a chronic persistent inflammatory process [2]. Tobacco smoking has been proven to be the predominant environmental factor for COPD. However, only approximately 15% of smokers develop airway obstruction [3,4]. This phenomenon, together with the familial clustering in patients with COPD [5], suggested that genetic factors might play an important role in COPD development.

Numerous studies have demonstrated that the balance between the serine proteases and serine protease inhibitors (SERPINs) is important in maintaining the matrix biology of the lung cells [6-8] and an imbalance between these two factors has been a long-standing hypothesis to explain lung destruction in emphysema during developing COPD [7]. However, in the SERPINs family, only *SERPINA*1 (α1-antitrypsin) has been confirmed to be a genetic risk factor for this disease so far. In addition, the mutant protease inhibitor (Pi) Z homozygote of the *SERPINA*1 gene, which can increase individual susceptibility to COPD, is rare across worldwide populations (0.001% – 4.5%), especially in Asians (< 0.004%), and accountable for only 2% COPD patients [9]. All of these observations suggested that other members of this gene family may also be involved in the onset of COPD and deserve further investigation.

Serine protease inhibitor E2 (*SERPINE2*, NM_006216) is one member of the SERPINs family. Due to the elevated expression of *SERPINE*2 in murine lung development, and consistent correlations with COPD-related phenotypes in human lung tissues, *SERPINE2 *was supposed to be a novel candidate gene for COPD [10]. Further investigation identified some SNPs in *SERPINE2 *that might confer individual susceptibility to COPD by influencing matrix metalloproteinase pathways [10,11]. However, this issue was questionable since these associations have not yet been verified [12]. To untangle this controversy and illustrate the relationship between *SERPINE2 *and COPD, additional evidence from populations with different environments and/or genetic backgrounds is warranted. Moreover, all of the above studies focused only on Caucasians, it remains unclear whether their conclusions also applied to East Asian populations, whose genetic background has been proven to be strikingly different from Caucasian.

In the present study, the five putative causal SNPs in *SERPINE2 *were genotyped in a case-control population from southwest Han Chinese and their potential correlations with COPD were evaluated. Furthermore, the relationship between *SERPINE2 *and COPD severity was also investigated. Our results would help to illustrate the genetic contribution to this disease.

## Methods

### Study population

327 unrelated patients with COPD and 349 healthy smokers were recruited from the First Affiliated Hospital of Kunming Medical College (Kunming, China). All smokers belonged to the Han nationality to minimize the potential sampling bias due to population stratification. COPD patients were diagnosed based on the results from multiple examinations, including the ratio of forced expiratory volume in one second/forced vital capacity (FEV_1_/FVC ratio < 70%), and FEV_1 _< 80% predicted, according to the Global Initiative for Chronic Obstructive Lung Disease criteria [2]. Based on the pulmonary function, the patients were further classified into two subgroups: mild/moderate COPD (FEV_1 _> 50% pred); and severe/very severe COPD (FEV_1 _≤ 50% pred), to evaluate whether *SERPINE2 *contributed to the disease severity. The healthy smokers exhibited normal pulmonary function (FEV_1_/FVC > 70% and FEV_1 _> 80% pred) and a smoking history ≥ 10 pack-yrs, and were excluded from the possibility of COPD by a Chest CT. This study was approved by our institutional ethics committee, and informed consent was obtained from all participants. The detailed information of patients and healthy smokers was listed in Table 1.

**Table 1:** The demographic character of study subjects

	Cases	Controls
Subjects (n)	327	349
Male (%)	80.1	71.9
Age	71.4 ± 9.1 *	67.0 ± 9.8 ^#^
Smoking (pack-yrs)	30.2 ± 6.7	29.2 ± 6.9
FEV_1_/FVC	45.3 ± 15.6	86.4 ± 5.8
FEV_1 _% pred	54.9 ± 18.9	94.9 ± 8.7
FEV_1 _> 50% pred	170	0
FEV_1 _≤ 50% pred	157	0

* data available for 210 patients, ^# ^data available for 208 subjects.

Data are presented as mean ± SD.

### Genotyping procedures

Genomic DNA was isolated from whole blood leukocytes by the conventional phenol-chloroform method.

Five SNPs in *SERPINE2 *(rs3795877, rs6747096, rs3795879, rs7579646 and rs840088) were genotyped by PCR amplification and direct resequencing. Since three of them, rs3795877, rs6747096 and rs3795879, showed strong linkage disequilibrium (LD) but yielded different results [10,12], we included all three instead of one tag SNP for genotyping, with the attempt to investigate whether it was also the case in our cohort. PCR was performed in a 50 μL reaction mixture consisting of 250 ng genomic DNA, 5 μL 10 × PCR Buffer (20 mM Tris-HCl pH 8.3, 500 mM KCl, 15 mM MgCl_2_), 2 μL dNTP mixture, 1 μL of each primer (10 μM) and 1 U Taq DNA polymerase. Reaction conditions for PCR were as follows: 95°C for 5 minutes, 35 cycles consisting of 94°C for 1 minute, ~56°C (detailed annealing temperature for each amplicon shown in Additional file 1) for 1 minute, and 72°C for 1 minute, and finally with an incubation at 72°C for 5 minutes.

The PCR products were separated by electrophoresis on a 1.5% agarose gel, recovered by column kit (Watson Biotechnologies Inc., China), and resequenced directly by BigDye™ Terminator v3.1 Cycle Sequencing kit and ABI PRISM 3730 sequencer (Applied Biosystems Inc., USA). The known positive and negative controls were included in the sequencing process and all samples were sequenced on both strands. The resulting sequences were analyzed with DNAStar software Package (DNASTAR Inc., USA).

### Statistical analysis

Age, smoking history, and pulmonary function data were displayed as mean ± SD. Hardy-Weinberg equilibrium was tested using a goodness-of-fit χ^2 ^test with one degree of freedom. The frequencies of each SNP between patients and controls, and between different disease groups (mild/moderate *vs*. severe/very severe) were compared by two-tailed Chi-square tests. All statistical tests were performed in SPSS 13.0 (SPSS Inc., USA). Pairwise LD between each SNP was evaluated by a maximum likelihood method to infer phase for dual heterozygotes, and quantified as r^2^. The individual haplotypes composed of the five SNPs were estimated from genotype data using the PHASE program (v.2.0) [13]. Odds ratios (OR) and 95% confidence intervals (CI) were also calculated to assess the relative disease risk. Significance was accepted when *P *(Probability) value was < 0.05.

## Results

The genotype and allele frequencies of the five *SERPINE2 *SNPs in patients with COPD and in healthy smokers were listed in Table 2. All five SNPs were in Hardy-Weinberg equilibrium in both case and control group (*P *> 0.1). Three SNPs (rs3795877, rs6747096 and rs3795879) were in complete LD, which was consistent with HapMap data (CEU, CHB and JPT populations), and thus presented the identical pattern in genotype distribution. The genotype frequencies of these three SNPs were 1.8%, 22.5%, and 75.7% in patients while 2.0%, 21.8%, and 76.2% in controls, for homozygote of minor allele, heterozygote, and homozygote of major allele, respectively. Due to the similar frequencies, no significant difference was observed (Heterozygote OR 1.03, 95%CI 0.72–1.48, Homozygote OR 0.92 95%CI 0.30–2.77, *P *= 0.97). Consistent with genotype distribution, a nearly identical minor allele frequency, 13.0% in cases and 12.9% in controls respectively, was obtained (*P *= 0.96). The other two SNPs, rs7579646 and rs840088, showed a similar pattern to the first three (see Table 2). Since COPD was observed mainly in males, we excluded 64 women from the case cohort and 98 women from the control population and consequently only males were considered for further analysis. The results, however, were still not significant (data not shown).

**Table 2:** Genotypes and alleles distribution in patients with COPD and healthy smokers

	Case (%)	Control (%)	OR [95% CI]	*P *value		
				
				Allele test	Genotype test	HWE*
rs7579646						
A	17.6	18.2		0.77	0.62	0.60
G/G	69.4	67.3	1.00			
G/A	26.0	28.9	0.87 [0.62–1.23]			
A/A	4.6	3.7	1.19 [0.56–2.56]			
rs840088						
T	43.0	43.3		0.91	0.91	0.22
C/C	33.3	33.8	1.00			
C/T	47.4	45.8	1.05 [0.75–1.48]			
T/T	19.3	20.3	0.96 [0.63–1.47]			
rs3795877						
C	13.0	12.9		0.96	0.97	0.57
T/T	75.8	76.2	1.00			
T/C	22.3	21.8	1.03 [0.72–1.48]			
C/C	1.8	2.0	0.92 [0.30–2.77]			
rs6747096						
G	13.0	12.9		0.96	0.97	0.57
A/A	75.8	76.2	1.00			
A/G	22.3	21.8	1.03 [0.72–1.48]			
G/G	1.8	2.0	0.92 [0.30–2.77]			
rs3795879						
A	13.0	12.9		0.96	0.97	0.57
G/G	75.8	76.2	1.00			
G/A	22.3	21.8	1.03 [0.72–1.48]			
A/A	1.8	2.0	0.92 [0.30–2.77]			

SNP = Single nucleotide polymorphism, OR = Odds ratio, CI = Confidence interval.

*P-value from test for Hardy-Weinberg equilibrium in controls

In order to investigate whether *SERPINE2 *SNPs contributed to the severity of COPD, we also compared the frequencies of these five SNPs in mild/moderate and severe/very severe subgroups (shown in Table 3). The three SNPs in complete LD (rs3795877, rs6747096 and rs3795879) presented a similar pattern in minor allele frequency: 13.5% in the mild/moderate group while 12.4% in the severe/very severe one respectively, suggesting that there was no significant difference (*P *= 0.67). The genotype distribution of these three SNPs also failed to yield a significant result (*P *= 0.86). The other two SNPs (rs7579646 and rs840088) displayed a similar result (see Table 3). In both total cohort and subgroup analysis, these results did not change after adjusting for multiple significant testing by Bonferroni correction or false discovery rate (FDR).

**Table 3:** Distribution of genotypes and alleles in copd subgroups

	mild/moderate (%)	severe/very severe (%)	OR [95% CI]	*P *value	
				
				Allele test	Genotype test
rs7579646					
A	17.4	17.8		0.87	0.63
G/G	70.6	68.2	1.00		
G/A	24.1	28.0	0.83 [0.50–1.37]		
A/A	5.3	3.8	1.34 [0.46–3.87]		
rs840088					
T	45.0	40.8		0.27	0.26
C/C	29.4	37.6	1.00		
C/T	51.2	43.3	1.51 [0.92–2.47]		
T/T	19.4	19.1	1.30 [0.70–2.42]		
rs3795877					
C	13.5	12.4		0.67	0.86
T/T	74.7	77.1	1.00		
T/C	23.5	21.0	1.15 [0.68–1.95]		
C/C	1.8	1.9	0.95 [0.19–4.81]		
rs6747096					
G	13.5	12.4		0.67	0.86
A/A	74.7	77.1	1.00		
A/G	23.5	21.0	1.15 [0.68–1.95]		
G/G	1.8	1.9	0.95 [0.19–4.81]		
rs3795879					
A	13.5	12.4		0.67	0.86
G/G	74.7	77.1	1.00		
G/A	23.5	21.0	1.15 [0.68–1.95]		
A/A	1.8	1.9	0.95 [0.19–4.81]		

SNP = Single nucleotide polymorphism, OR = Odds ratio, CI = Confidence interval.

To further assess whether any of the SNPs influence COPD onset, logistic regression analysis was performed in 418 subjects (210 cases and 208 controls) with full records in age, sex, smoking history and all genotyping data. In this logistic regression analysis, FEV_1 _(% pred) that classified the case and control was regarded as the outcome variable, while sex and smoking history were covariates with allele count (0/1/2) for each SNP genotype. Our results indicated that men were more likely to develop COPD than women, and COPD was more prevalent in elderly persons, which was in agreement with the previous study [14]. However, others factors, including the genetic variants, did not influence FEV_1 _(% pred) (data not shown).

We also calculated the pairwise LD for the five SNPs. There was complete LD among the three SNPs: rs3795877, rs6747096 and rs3795879 (r^2 ^= 1). The other two SNPs (rs7579646 and rs840088) were in low LD both with the three abovementioned ones (r^2 ^= 0.478 and 0.133, respectively) and within themselves (r^2 ^= 0.288). In addition to single SNP analysis, a combination analysis for these SNPs was also performed. The result indicated that there were four major haplotypes with a frequency above 3% in our samples, and their frequencies varied from 7.1% to 54.6% in patients (shown in Table 4). No haplotypes were observed to affect the OR of COPD significantly (ORs range 0.87–1.19; *P *> 0.1), which was consistent with the results of single SNP analysis.

**Table 4:** The distribution of *SERPINE2 *haplotypes in COPD and control groups

Haplotype *	Cases (%) ^#^	Controls (%) ^#^	OR [95%CI] ^&^	*P *value
G C T A G	356 (54.4)	395 (56.5)	0.95 [0.77–1.19]	0.67
G T T A G	166 (25.4)	170 (24.3)	1.08 [0.84–1.39]	0.54
A T C G A	68 (10.4)	84 (12.0)	0.87 [0.62–1.22]	0.42
A T T A G	47 (7.2)	43 (6.2)	1.19 [0.78–1.82]	0.43
others	17 (2.6)	6 (1.0)		
total	654 (100.0)	698 (100.0)		

OR = Odds ratio, CI = Confidence interval

*Composed of five SNPs: rs7579646, rs840088, rs3795877, rs6749096, and rs3795879; ^# ^Data are presented as number of chromosome (%); ^&^OR value just for the major haplotypes (haplotype frequency > 3%).

## Discussion

Since COPD is a complex disease caused by multiple genetic and environmental factors, it is essential to obtain more data on different populations to elucidate the role of the *SERPINE2 *in COPD. In the present study, we provided new genotyping data of *SERPINE2 *SNPs in the Han Chinese population, but failed to obtain any significant association between SNPs, including both genotype and haplotype, and the disease phenotype. Our results were not consistent with DeMeo's [10], but confirmed the conclusion of Chappell et al. [12]. Interestingly, a recent paper from the DeMeo group also failed to replicate their original result on these five SNPs in another population either [11]. It was also worth mentioning that several other SNPs, such as rs6734100, which failed to present correlation with COPD in original study [10], showed the most significant association in Zhu et al. [11] and deserved further investigation.

When conflicting results among different studies of this kind occur, common explanations include population stratification, genetic heterogeneity, phenotypic heterogeneity, and statistical power. Generally, genetic heterogeneity is often a factor to explain this conflict [15]. Considering that the genetic background of Caucasians is strikingly different from that of East Asians, we cannot rule out the possibility that the conflict may just reflect the different pathology of this disease in different ethnic populations.

Sometimes a small sample size may bring some bias into association studies [15]. To investigate whether our sample size was sufficient to detect genetic determinants of minor effect, we assessed the power of our sample size by using the genetic power calculator [16]. With the 2.0% disease prevalence in the Kunming population [17] and α = 0.05, for a variant with 0.1–0.4 minor allele frequency in an additive model, our sample size provided 60% power to detect a genetic relative risk (or OR) of 1.5, while for a genotypic OR of 1.75, the power increased to ≥ 80%.

In addition to these, it was worth noting that some results of statistical analyses were not interpreted decently in DeMeo et al. [10]. Since as many as 48 SNPs were genotyped and investigated in that study, over two SNPs would yield a significant result by chance given a random distribution and a threshold of 0.05. To reduce any false positive effect and obtain a reasonable result, some corrections, such as Bonferroni correction or FDR, would be crucial, and would definitely lower the significance level dramatically. Since the original genotyping data of DeMeo et al. [10] was not available and thus the FDR could not be calculated, Bonferroni correction was used, despite the fact that this method was somewhat conservative. As a result, the original significance level, 0.05, should be divided by 16, since at least 16 independent LD blocks exist among their 48 SNPs according to HapMap data in the Caucasian population (LD pattern not shown) and consequently, a much stricter one, 0.003, should be applied for assessing significance. Under this circumstance, most *P *values in DeMeo et al.' result would be beyond this threshold and thus these SNPs should not be considered to be positive, which was in agreement with Chappell et al. [12] and our results.

Another concern is about the potential mechanism how these SNPs in *SERPINE2 *could play a role in COPD susceptibility. Since these five SNPs are located in the first three introns and over-expression of *SERPINE2 *was supposed to be correlated with COPD [10], it was reasonable to hypothesize that these SNPs might alter the *SERPINE2 *expression in tissues, given these five SNPs really correlated with COPD susceptibility. To test this hypothesis, we retrieved the gene expression data of *SERPINE2 *from HapMap lymphoblastoid cell line [18] and HapMap genotype data [19], and performed a linear regression analysis on these five SNPs in four HapMap populations. The three SNPs in strong LD, rs3795877, rs6747096 and rs3795879, did not present correlation with gene expression, except in the JPT population (r^2 ^= 0.32, *P *= 0.0012). Similarly, the remaining two SNPs (rs7579646 and rs840088) also failed to show any correlation in all four HapMap populations, except for rs840088 in the CHB population (r^2 ^= 0.15, *P *= 0.027). Considering that this relationship was observed randomly with two SNPs in two populations and that expression data was only available for the lymphoblastoid cell line, which represents only one tissue type, it was far from drawing any conclusion on whether these SNPs could regulate the expression of *SERPINE2*. More studies on gene expression, especially expression in lung cells or a cell line with lung derivation, are necessary to clarify this issue.

## Conclusion

In summary, our study was the first report to investigate the association between *SERPINE2 *SNPs and COPD in an East Asian population. The negative results we obtained, in combination with the available evidence in the literature, suggest that it is still far from reaching a consensus and more studies should be surveyed.

## Abbreviations

CI: Confidence interval; COPD: Chronic obstructive pulmonary disease; FDR: False discovery rate; LD: Linkage disequilibrium; OR: Odds ratio; Pi: Protease inhibitor; *SERPINE2*: Serine protease inhibitorE2; SERPINs: Serine protease inhibitors; SNP: Single nucleotide polymorphism.

## Competing interests

The authors declare that they have no competing interests.

## Authors' contributions

LZ contributed to the design of this study, carried out the molecular genetic studies, and drafted the manuscript. WPF and LMD undertook the data collection and contributed to the editing of the manuscript. CS assisted with the statistical analysis and the manuscript composing. YPZ contributed to the conception and the design of the study and critically revised the manuscript. All authors read and approved the final manuscript.

## Pre-publication history

The pre-publication history for this paper can be accessed here:



## Supplementary Material

Additional file 1 **online supplementary material-final**. Supplemental material Click here for file
