# Supplementary material for: Absence of association between SERPINE2 genetic polymorphisms and chronic obstructive pulmonary disease in Han Chinese: a case-control cohort study
Source: BMC Med Genet. 2009 Jul 16;10:66. doi: 10.1186/1471-2350-10-66 (PMC2719615; doi:10.1186/1471-2350-10-66)
Supplement: Additional file 1 — online supplementary material-final. Supplemental material [file 1471-2350-10-66-S1.doc]

**Online supplementary material**

**Table** 1 Primers and annealing temperatures in current study

| SNPs | Allele | Positions in the gene | Locations in chromosome ^*^ | Primers (5’---3’) ^&^ | Anealing Temp (°C) |
| --- | --- | --- | --- | --- | --- |
| rs7579646 | G/A | Intron1 | 224,608,940 | F--TCACCAATATGAAGCGAGTCC | 55 |
|  |  |  |  | R--CAAGGCCAGCGTGACTAGAG |  |
| rs840088 | C/T | Intron1 | 224,608,088 | F--TTGCTTATAGTGGCGTTCT | 55 |
|  |  |  |  | R--GGGCAGTAGTCAGGCTTGTAG |  |
| rs3795877 | C/T | Inton2 | 224,574,421 | F--CTTGGGGCTTGGGGTCAGTCTCT | 60 |
|  |  |  |  | R--AAATTTCCATCAGGTCAGTCCACA |  |
| rs6747096 | A/G | Exon3 | 224,571,086 | F--ATGCCTCTGAAATTGAAGTGC | 60 |
|  |  |  |  | R--AGTGGAGGGGCTGGATTC |  |
| rs3795879 | A/G | Intron3 | 224,571,065 | F--ATGCCTCTGAAATTGAAGTGC | 60 |
|  |  |  |  | R--AGTGGAGGGGCTGGATTC |  |

SNPs = Single-nucleotide polymorphisms

^*^ referred to NCBI build 36. ^&^ F for the forward primer whereas R for the reverse primer.
